# Immunotherapy for Triple-Negative Breast Cancer

**DOI:** 10.3390/pharmaceutics13122003

**Published:** 2021-11-25

**Authors:** Yifeng Cao, Chuyang Chen, Yi Tao, Weifeng Lin, Ping Wang

**Affiliations:** 1 College of Pharmaceutical Sciences, Zhejiang University of Technology, Hangzhou 310014, China; 2111907052@zjut.edu.cn (C.C.); taoyi1985@zjut.edu.cn (Y.T.); 2 Department of Molecular Chemistry and Materials Science, Weizmann Institute of Science, Rehovot 76100, Israel; lin.weifeng@weizmann.ac.il

**Keywords:** TNBC, immunotherapy, immune checkpoint, nanocarrier, drug delivery, combinational immunotherapy

## Abstract

Triple-negative breast cancer (TNBC) is characterized by extensive tumor heterogeneity at both the pathologic and molecular levels, particularly accelerated aggressiveness, and terrible metastasis. It is responsible for the increased mortality of breast cancer patients. Due to the negative expression of estrogen receptors, progesterone receptors, and human epidermal growth factor receptor 2, the progress of targeted therapy has been hindered. Higher immune response in TNBCs than for other breast cancer types makes immunotherapy suitable for TNBC therapy. At present, promising treatments in immunotherapy of TNBC include immune checkpoints (ICs) blockade therapy, adoptive T-cell immunotherapy, and tumor vaccine immunotherapy. In addition, nanomedicines exhibit great potential in cancer therapy through the enhanced permeability and retention (EPR) effect. Immunotherapy-involved combination therapy may exert synergistic effects by combining with other treatments, such as traditional chemotherapy and new treatments, including photodynamic therapy (PTT), photodynamic therapy (PDT), and sonodynamic therapy (SDT). This review focuses on introducing the principles and latest development as well as progress in using nanocarriers as drug-delivery systems for the immunotherapy of TNBC.

## 1. Immunotherapy in Triple-Negative Breast Cancer (TNBC)

Cancer constitutes the largest public health problem in the world. According to the data released by the American Cancer Society (ACS), there are about 4950 patients diagnosed with cancer and 1662 dying of it every day; Among them, breast cancer is the most popular female cancer type, which is estimated to be nearly 30% of the new cases and the death rate of which is as high as 15% in 2020 [1]. According to the expression of estrogen receptors (ER), progesterone receptors (PR), and human epidermal growth factor receptor 2 (HER2), breast cancer is defined as three major subtypes: hormone-receptor (HR) positive, HER2-enriched, and triple-negative breast cancer (TNBC). TNBC accounts for 15–20% of all breast cancers, particularly those in young women [2]. Compared with the other subtypes, TNBC does not respond well to hormonal therapy or medicines targeting HER2 protein receptors. It shows higher drug resistance and tumor heterogeneity and aggressiveness, and is often accompanied by lung or brain metastasis. Lacking therapeutic target is the main reason challenging the effective treatment of TNBC. Once the tumor metastasizes, the median overall survival of TNBC patients is only 12 to 18 months due to the limited therapeutic window [3]. Additionally, TNBC contains more immune cells [4], and is easily recognized by the immune system due to its high genetic instability and tumor mutational burden, making it one of the tumor types suitable for immunotherapy intervention [5]. Although there are limited options for its treatment, TNBC is the most immunogenic subtype of breast cancers. The robust antitumor responses of immunotherapy in hematologic and solid malignancies bring hope to TNBC patients [6].

Changes in the tumor microenvironment (TME), including tumor cell proliferation, tumor metastasis, tumor recurrence, and tumor resistance, play a critical role in the progression of tumors as well as in their response to treatment and prognosis. In fact, the success of immunotherapy links the TME with immunity [7,8]. In particular, tumor immune microenvironment (TIME) composed of various immune cells has also attracted much attention and exhibits significant importance to immunotherapy. Immune cells, scattered in the tumor center and infiltrating edge or adjacent tertiary lymphoid tissue, can be roughly divided into immunosuppressive cells and immune effector cells [9,10]. Similar to many other cancers, the antitumor immune killing effect in TNBC is performed by cytotoxic T cells CD8+ and helper T cells CD4+ [10]. Regulatory T cells (Tregs) is a major group of immunosuppressive cells, characterized by elevated Foxp3 expression and tumor-associated macrophages [11]. They inhibit the immune function mediated by CD8+ and CD4+ T cells by secreting TGF-β, IL-10, and IL-35 in the process of immune escape. Meanwhile, they can kill T cells directly through producing granzymes and perforin [12]. When Tregs dominate the immune function of tumor, immune escape would occur [13].

**Figure 1:**
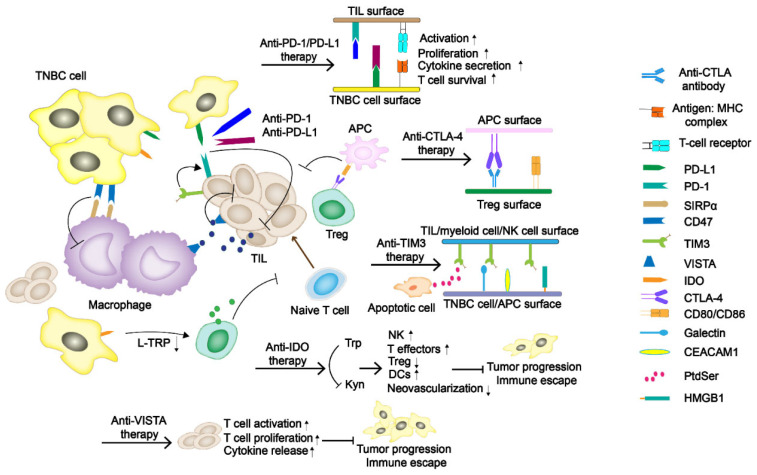
The regulatory mechanism of immune checkpoints (ICs) in TNBC tumor progress. The programmed cell death-1/programmed death-ligand 1 (PD-1/PD-L1) and cytotoxic T-lymphocyte-associated antigen-4 (CTLA-4) have been the primary immune checkpoint blockades. Some potentially new immune ICs, such as T-cell immunoglobulin and mucin domain-containing protein 3 (TIM-3), indoleamine 2,3-dioxygenase (IDO), as well as V domain Ig suppressor of T-cell activation (VISTA), are also demonstrated in the figure.

Immune checkpoints (ICs) are molecules playing a protective role similar to brakes in the immune system. It can prevent inflammation damage and autoimmune diseases caused by excessive activation of T cells (Figure 1). Tumor cells use human immune system to over-express immune checkpoint molecules to inhibit the response of the human immune system and to escape immune surveillance and killing [14,15]. In particular, programmed cell death protein-1 (PD-1) and cytotoxic T-lymphocyte-associated protein-4 (CTLA-4), which weaken the immune function mediated by T cells, are of great importance to tumor immunosuppression [16]. More potential checkpoints have been discovered, and their value in immunotherapy is gradually being explored. Meanwhile, adoptive T-cell immunotherapy and tumor vaccine are also constantly being studied as promising treatments.

### 1.1. Immune Checkpoint Blockades PD-1/PD-L1 and CTLA-4

Immune checkpoint blockades PD-1/PD-L1 and CTLA-4 are currently the primary and most widely studied immunotherapy agents (Figure 1). PD-1 is a member of the CD28 superfamily and is mainly expressed in activated T and B lymphocytes, natural killer (NK) cells, and myeloid cells. The structure of PD-1 includes an extracellular immunoglobulin variable region (IGV), a hydrophobic transmembrane domain, and an intracellular domain. The tail of the intracellular region contains the immune receptor tyrosine-based inhibitory motif (ITIM) and the immune receptor tyrosine-based switch motif (ITSM) [17]. PD-L1 and PD-L2 are two ligands of PD-1. The inhibitory signals often appear upon PD-1/PD-L1 binding, tyrosine phosphorylation in ITSM causes dephosphorylation of downstream protein kinases Syk and PI3K, hinders downstream pathway activation, and inhibits transcription and translation of genes and cytokines required for T-cell activation. Studies have shown that PD-L1 expression is positive in about 20% of the TNBC patients, which is significantly higher than that in non-TNBC patients [18]. CTLA-4 is a transmembrane protein exclusively expressed on T cells and Tregs in tumor infiltrating lymphocytes (TILs). It has a similar domain structure to CD28 (sharing 31% identity) and binds to B7.1 (CD 80) and B7.2 (CD 86) with higher affinity [19]. Moreover, its engagement on Tregs could strengthen the suppressive effect.

Currently, PD-1/PD-L1 inhibitors are mainly monoclonal antibodies (mAbs) and some small molecules [20]. For example, pembrolizumab and emiplimab are humanized IgG4κ monoclonal antibodies binding to PD-1 [21], nivolumab is an all humanized genetically engineered monoclonal antibody against PD-1 [22,23], and atezolizumab and durvalumab are both humanized IgG1κ type monoclonal antibodies against PD-L1 [24]. There are many ongoing clinical trials in the treatment of TNBCs with mAbs (Table 1). Immune checkpoint blockade therapy has been proved for the treatment of multiple types of cancer; however, none of them has been approved for the treatment of TNBC so far. Some problems came along with the clinical trials. Demaria et al. [25] concluded that CTLA-4 antibody monotherapy could not inhibit the growth of metastatic tumor in mouse breast cancer cell 4T1. In the randomized phase II trial NCT02519322, grade III adverse events occurred in 8% of patients treated with nivolumab monotherapy and as high as 73% of patients treated with both nivolumab and ipilimumab [26]. In the phase II clinical trial NCT02536794, durvalumab combined with tremelimumab were administered, but was finally terminated due to objective response rate (ORR) did not match the required criteria [27]. Fortunately, the KEYNOTE-012 trial in Phase Ib and KEYNOTE-086 trial in phase II both revealed that pembrolizumab had controllable safety and persistent antitumor activity in TNBC with PD-L1 positive expression [28,29].

### 1.2. T-Cell Immunoglobulin Domain and Mucin Domain-3 (TIM-3)

TIM-3, also known as HAVcr2 or CD366, is a type I cell-surface glycoprotein consisted of an amino-terminal immunoglobulin variable domain (V domain) with five noncanonical cysteines, a mucin-like stalk, a transmembrane domain, and an intracellular cytoplasmic tail [31]. It contains four different ligands, including galectin-9 (Gal-9), phosphatidylserine (PtdSer), carcinoembryonic antigen-related cell adhesion molecule-1 (CEACAM-1), and high mobility group protein B-1 (HMGB-1) [32]. TIM-3 is associated with tumor immune regulation and autoimmune diseases. Byun et al. [33] demonstrated that TIM-3 expression is a positive prognostic factor in TNBC. Due to the diversity of receptors causing the binding form to change under different situations, whether TIM-3 acts as a costimulatory receptor or a co-inhibitory receptor has not been fully determined. However, a recent study shows that TIM-3 mostly plays its role as an inhibitory receptor [34].

### 1.3. Indoleamine 2,3-Dioxygenase (IDO)

IDO is a rate-limiting enzyme in the catabolism of essential amino acid tryptophan (Trp) to kynurenine (Kyn). The downstream signal transduction of IDO includes the changes of general control non-derepressible-2 (GCN2), mammalian target of rapamycin (mTOR) and aryl hydrocarbon receptor (AhR) [35]. Research has proved that with the decrease of Trp followed by uncharged Trp tRNA accumulation, the GCN2 would be activated. Then, phosphorylation of eukaryotic initiation factor-2α (eIF2α) induced by GNC2 would inhibit the proliferation of effector T cells. Moreover, its metabolite Kyn can bind to AhR, leading to an increase in the number of Tregs. The suppression of mTOR and the increase of IL-6 secretion are both reasons why IDO exerts its immunosuppressive effect [36]. In breast cancer therapy, it is closely related to poor prognosis and increased microvessel density [37]. Asghar et al. [38] conducted a study on 100 female breast cancer patients in Pakistani (including triple-negative and non-triple-negative ones), linking the expression of IDO with median overall survival, proving that IDO plays a pivotal role in TNBCs. The overall survival of patients with low IDO expression is about 91 ± 41.9 months, which is much higher than the intermediate and high levels, 50 ± 4.4 and 24 ± 10.1 months, respectively. Because of the potential effect of IDO in immunotherapy, some of the IDO inhibitors, including Epacadostat, BMS986205, PF-06840003, Navoximod, Indoximod, NLG802, and LY3381916, are under the latest ongoing clinical trials [39].

### 1.4. V Domain Ig Suppressor of T-Cell Activation (VISTA)

VISTA, also referred to as PD-1H, is a newly discovered negative immune checkpoint related to immunotherapy resistance. VISTA is a type I transmembrane protein consisting of an N-terminal IgV domain, a stalk of about 30 amino acids, a transmembrane domain, and a cytoplasmic tail of 95 amino acids. Its molecule shares sequence homology with PD-L1 and PD-L2 [40,41]. Similar to PD-1, VISTA also serves as a negative regulatory agent for T cells by suppressing their activation, proliferation, and cytokine release. In breast cancer, it is expressed on TILs, macrophages, and other immune cells. Xue et al. [42] found that VISTA expression was higher in CD68+ tumor-associated macrophages (32.58%), CD4+ T cells (4.97%), CD8+ cytotoxic T cells (4.48%), and CD20+ B cells (1.46%). Gao et al. [43] proved that treating prostate cancer with ipilimumab can lead to a compensatory up-regulation of VISTA, indicating that VISTA may be related to the development of resistance to immune checkpoint blocking therapy. However, Cao et al. [44] evaluated the expression of VISTA in a cohort of 254 untreated TNBC patients, and found that VISTA was expressed in 87.8% (223/254) and 18.5% (47/254) of the immune cells and tumor cells, respectively. Meanwhile, the expression of VISTA in ICs is positively correlated with some TILs, especially CD4+ TIL. The information confirms the regulatory role of VISTA in antitumor immunity, but it has not been developed as a negative immune checkpoint so far.

### 1.5. Adoptive T-Cell Immunotherapy

The adoptive T-cell immunotherapy starts with isolating T cells, which are afterwards genetically modified to express CRA, followed by proliferation, then are reinjected to patients. The injected T cells combine with specific antigens, and ultimately eliminate targeted tumor cells [45]. Moreover, using genetic engineering technology, two types of engineered T cells, T cells with T-cell receptor (TCR) and Chimeric antigen receptor T-cell (CAR-T), were given higher recognition. CAR-T immunotherapy, started in the late 1980s, has been proved to play a key role in CD19 positive hematological malignancies [46]. CARs are synthesized receptors which consist of extra- and intracellular domains: the extracellular part is single chain fragment variable (scFv) domain composed of specific antibody, while the intracellular domain contains CD3ζ and CD3ζ co-stimulate with CD28, ICOS, 4-1BB (CD137), CD27, or OX40 signals domain. The main advantage of CAR-T technique is that it can recognize cancer cells without the presence of major histocompatibility complex (MHC) antigen.

To develop an effective CARs therapy for TNBCs, it is necessary to select a desired tumor cell-surface antigen, which can be express stably in most tumor cells, and has high tumor specificity [47]. For this purpose, Song et al. [48] designed a new folate receptor α (FRα)-specific CAR-T, which was composed of MOv19 scFv and CD8a hinge in extracellular region, and of CD27 costimulatory domain and CD3ζ signaling domain in the intracellular region. The FRα-specific CAR-T cells show a more robust immune effect in TNBCs with FRα protein overexpression. Zhou et al. [49] generated the MUC28z CAR-T cell consisted of TAB004 scFv coupled with CD28 and CD3ζ, and demonstrated that MUC28z CAR-T cells have high tumor antigen specificity, and refrained recognition of normal tissues. Moreover, it is found that MUC28z CAR-T cells can lyse TNBCs and reduce the tumor growth both in vitro and in vivo. Moreover, the growth factor receptor (EGFR) is a potential tumor surface antigen, and EGFR-specific CAR-T has strong cytotoxicity. However, the emergence of drug resistance is an urgent problem. Lin et al. [50] combined EGFR-specific CAR-T with THZ1, a CDK7 inhibitor, which together demonstrated a good effect on TNBCs proliferation, tumor metastasis and suppressed immune resistance in mice. Identically, Stuber et al. [51] combined ROR1-specific CAR-T cells to SD-208, a TGF-β inhibitor, to weaken the immunosuppressive effects in therapy.

### 1.6. Tumor Vaccine Immunotherapy

In contrast to the traditional concept of vaccination, the definition of vaccine in modern medicine is not only limited to prevention of diseases, but expanded to target disease-specific antigens for the treatment of ongoing diseases. In the development of immune vaccines, tumor-associated antigens and delivery technology are the main considerations. The neoantigens is produced when gene coding contained non-synonymous mutations and only expressed in tumors [52]. However, tumor-associated antigens can be expressed in tumors and normal tissues at the same time. Studies have shown that neoantigens have a stronger affinity to human leukocyte antigen (HLA) and T-cell receptors, and are not limited by central tolerance and autoimmune problems [53] Therefore, it has been regarded as one of the most potential tumor treatment targets since its discovery, and is the key to the development of personalized immunization vaccines.

Immunization vaccine also offers a viable option in the treatment of TNBCs. In the study by Liu et al. [54], an mRNA-based vaccine encoding tumor antigen MUC1 was delivered to dendritic cells (DCs) in lymph nodes using a nano-delivery system, and anti-CTLA-4 antibodies were used in combination to exert antitumor effects. Among them, the nano-delivery system can enhance the stability, persistence and expression level of the vaccine, and the vaccine that enters the body plays a role by activating and expanding tumor-specific T cells. Compared with other groups, the combination treatment group has the strongest effect of inhibiting tumor growth. Pack et al. [55] isolated tumor membrane vesicles (TMV) form 4T1 tumor, and glycosylphosphatidylinositol (GPI) anchored form of immunostimulatory B7-1 (CD80) and IL-12 molecules were combined to these TMVs to prepare TMV vaccine. Compared with monotherapy, tumor-bearing mice administrated with combined treatment of vaccine and anti-CTLA-4 antibody exhibited significantly improved survival rate and reduced lung metastasis.

### 1.7. Immunotherapy-Involved Combination Therapies

In the treatment with immune checkpoint inhibitors (ICIs), though mAbs show certain therapeutic effect, the response rate is generally low. For instance, the response rate of pembrolizumab monotherapy in TNBC is only 5.3% [29]. To solve this problem, combination therapy, combining mAbs with various chemotherapeutics, such as nab-paclitaxel, epirubicin, cyclophosphamide, with radiotherapy, or with some cytokines, has been intensively used in clinic trials. Table 1 summarizes such clinical trials that are being recruited or in progress. For example, the ongoing clinical phase II trial NCT02730130 was designed to assess the efficacy and safety of pembrolizumab with radiotherapy. Results show that the obtained ORR and progression-free survival (PFS) were dramatically higher than those for pembrolizumab monotherapy, increased from 3% to 17.6% and 1.9 to 2.6 months, respectively [56]. As for the phase III clinical trial KEYNOTE-355 using combined pembrolizumab, nab-paclitaxel, carboplatin, and paclitaxel/gemcitabine the combination therapy resulted in a remarkable and clinically meaningful improvement in median PFS (4.1 months longer) [57]. Similarly, phase III trial Impassion130 showed that atezolizumab plus nab-paclitaxel would induce significantly longer PFS in TNBCs [58]. As for CTLA-4, Bernier et al. [59] demonstrated that combining DZ-2384 with CTLA-4 antibody could slow down tumor growth and increase overall survival rate. Li et al. [60] demonstrated that compared with monotherapy in mice, matrix metalloproteinase inhibitor plus CTLA-4 antibody could delay tumor growth and reduce distant metastases.

It is worth noticing that the combination therapy has been designed mostly for locally advanced or metastatic TNBCs. Neoadjuvant therapy, which has been used in the treatment of melanoma or colon cancer, is a promising strategy for early TNBC. The KEYNOTE-522 trial (NCT03036488, phase III) using a combination of pembrolizumab (MK-3475) and chemotherapy as adjuvant therapy for participants with early-stage TNBCs show that the rate of pathological complete response is significantly higher than in the placebo-chemotherapy group, even for patients with low PD-L1 expression [61].

## 2. Nanocarriers for the Immunotherapeutic Treatment of TNBC

The emergence of immunotherapy has shed light to the treatment of TNBC. However, due to the complex tumor microenvironment and complicated immunosuppressive mechanism, conventional drug administration methods are still limited to relatively low immune response and high adverse side effects. Thus, rational design of functional drug-delivery systems is necessary to improve drug targeting, control drug release, and obtain favorable pharmacokinetic behavior, enhanced drug absorption and more drug passing through biological barriers. In particular, using nanocarriers for the delivery of immune-responsive drugs can take advantage of the enhanced permeability and retention (EPR) effect, making more drug accumulate at the tumor site and a longer time circulation period [62,63,64]. The unique EPR effect and active targeting modification of drug-delivery systems play significant roles in the therapies for tumor in deep positions or metastasized. Therefore, nanocarrier-based immunotherapy may provide TNBC patients with safer and more effective treatment [65,66].

### 2.1. Nanomaterials for Delivering the Immunotherapeutic Agents of TNBC

**Figure 2:**
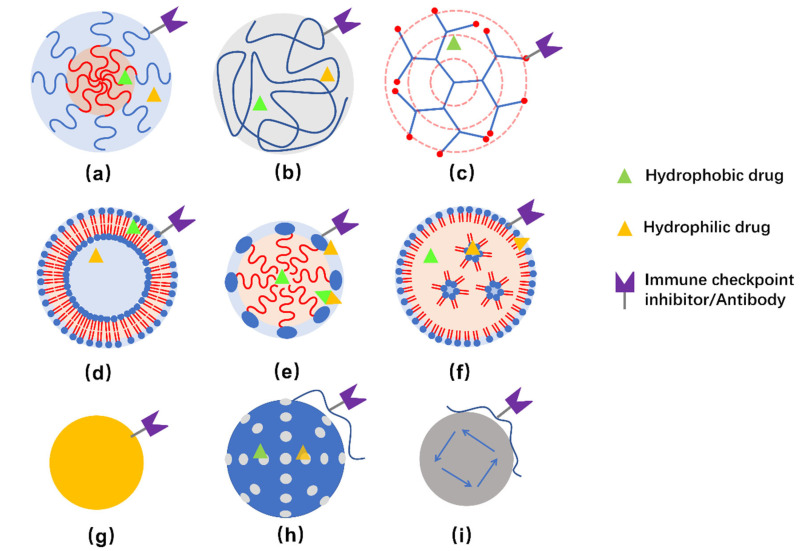
Schemes of structure of polymeric nanocarriers (**a**–**c**), lipid-based nanocarriers (**d**–**e**), and inorganic NPs (**g**–**i**). (**a**) polymeric micelle, (**b**) polymeric NP, (**c**) dendrimer, (**d**) liposome, (**e**) lipid emulsion, (**f**) lipid NPs, (**g**) Au-NPs, (**h**) silica NPs, and (**i**) magnetic NPs. Hydrophobic, hydrophilic, as well as amphiphilic drugs can be embedded in corresponding regions. Meanwhile, it is possible to conjugate immune checkpoint inhibitors and/or antibodies to the surface of nanocarriers for therapeutic and/or targeting purposes. Usually, hybrid NPs are developed to obtain multiple function or improved properties for delivering the drugs.

Commonly studied NPs for delivering immunotherapeutic drugs to treat TNBC include polymeric micelles, dendrimers, liposomes, inorganic NPs, and so on [67]. Illustration of some representative NPs are presented in Figure 2.

The physiochemical properties of polymers, such as charge, hydrophobicity/hydrophilicity, as well as the features of the polymeric NPs, include size, shape, and rigidity, can be tuned for encapsulating specific immune-responsive drugs and be designed to improve the endocytic uptake, biodistribution, and body clearance properties as well [68,69,70]. Methods for preparing nano-sized polymeric particles include polymeric micelles prepared by self-assembling of co-polymer consisting of hydrophobic and hydrophilic sections, polymeric NPs prepared by solvent evaporation, emulsification/solvent diffusion, nanoprecipitation or emulsification/reverse salting-out, as well as dendrimers prepared by repetitive addition of monomers initiated from a polyfunctional center [71,72,73]. Poly(lactic-co-glycolic acid) (PLGA) and polylactic acid (PLA) are biocompatible and biodegradable polymers approved by FDA in drug-delivery systems [74,75]. Dendrimers are composed of a hydrophobic core, which favors the encapsulation of hydrophobic molecules, and the outer surface that provides sites for functionalization.

Lipids are amphiphilic molecules composed of hydrophilic headgroup and hydrophobic tail(s). Lipid-based nanocarriers for drug delivery include solid lipid nanoparticles, vesicular liposomes, nano-emulsions/nano-micelles, as well as non-spherical ones, such as nanotubes. The configuration of the nanocarriers strongly depends on the packing parameter of the lipids [76]. Phospholipids, being the major components of cell membranes, have attracted particular attention as drug-carrier materials due to their good biocompatible, low toxicity and higher permeation. Liposomes prepared from aggregated synthetic phospholipid(s) or even directly extracted from tumor cells are preferred as drug-delivery carriers [77,78]. Similar to polymeric micelles, liposomes, lipid emulsions as well as lipid NPs are composed of both hydrophobic and hydrophilic regions, which can be used to encapsulate hydrophobic and hydrophilic drugs, respectively. Amphiphilic drugs may also be encapsulated at the hydrophilic-hydrophobic interface.

Inorganic NPs are robust frameworks allowing encapsulation and incorporation of one or more drugs or therapeutic molecules. Several inorganic NPs, including gold NPs (Au-NPs), mesoporous silica NPs, magnetic NPs, and carbon nanotubes, have been investigated as nanocarriers for drug delivery [79]. In addition to the loaded therapeutic agents, some NPs exert specific functions by themselves. For example, Au-NPs can induce cell death [80], and can also adsorb light in the NIR region and dissipate heat to the surroundings. As for magnetic nanoparticles (MNPs), they not only generate nonuniform magnetic fields which affect the morphology, differentiation and function of cells by generating magnetically induced mechanical forces [81], but also activate anti-cancer immune responses through their own immunomodulatory effects [82]. Inorganic NPs coated with polymers or lipid bilayers, forming core-shell structures that enable surface modification and show enhanced biocompatibility, have also been widely studied [83].

### 2.2. Nanocarriers for the Delivery of Immune Checkpoint Blockade Molecules

IC blockade is the primary strategy for TNBC immunotherapy, but the effect of monotherapy is modest. Increasing the dose or choosing a combination of multiple ICIs is commonly used to overcome this problem. However, the increase in drug toxicity has led to the suspension of many clinical trials. For instance, in the randomized phase II trial NCT02519322, grade III adverse events occurred in 8% of patients treated with nivolumab monotherapy and in as high as 73% of patients treated with both nivolumab and ipilimumab [26].

Combining more than one ICIs can exert more effects than using one ICI. Considering there are more expressed CD155 and PD-L1 in TNBC than in other types of breast cancers, Chen et al. [84] designed mPEG-PLGA-PLL (PEAL) NPs loaded with CD155 siRNA (siCD155) by a double-emulsion method followed by coated with a PD-L1 blockade, termed as P/PEAL_siCD155_, for immunotherapy of TNBC. In CD8+ TIL cells, CD155 can bind to different receptors, such as DNAM-1, TIGIT, and CD96 [85,86]. Based on the asynchronous expression of the above-mentioned receptors, this study shows that siCD155-mediated knockdown of CD155 by P/PEAL_siCD155_ can achieve spatiotemporal targeting of surface receptors and intracellular mRNA, making the antitumor effect take place in favorable periods, i.e., promoted CD155-mediated immune surveillance in the early stage and inhibited CD155-mediated immune evasion in the later stage. In the 4T1 TNBC tumor model, P/PEAL_siCD155_ showed surprising biocompatibility, specific targeting, along with efficient inhibition of TNBC tumor progression and metastasis [84].

Discovering more efficient molecules is another option overcoming the problems along with monotherapy. NPs made of 100% BMS-200, a small-molecular inhibitor of PD-1/PD-L1 interaction, has also been developed as potential alternatives to anti-PD-L1 monoclonal antibody (α-PD-L1) [87]. Compared with α-PD-L1, BMS-202 NPs show equivalent immunotherapy effect by inhibiting >90% primary and distant tumors, and possible superior tumor penetration. The approach of using NPs for the delivery of immune checkpoint blockade molecules may provide an alternative nanomedicine for treating metastatic or advanced TNBC.

### 2.3. Nanoparticles for the Delivery of Combination Therapy Agents

The monotherapies of TNBC, including chemotherapy, radiotherapy, and immunotherapy based on ICs, all demonstrate limited therapeutic effect. Based on the low pH levels, endogenous H_2_O_2_, overexpressed enzymes, and other specific factors in the TME of TNBCs, combination therapy using immunotherapy with other specific treatment, such as chemotherapy, photothermal therapy (PTT), photodynamic therapy (PDT), and sonodynamic therapy (SDT)*,* to overcome the insufficient efficacy by monotherapy and improve the therapeutic efficacy to TNBCs [88,89]. Combination therapy may also generate synergistic effect to the treatment of TNBC [90].

#### 2.3.1. Immunotherapy Combined with PTT

PTT is a process that converts light energy into heat to induce thermal ablation of the tumor. Near-infrared region (700–1350 nm) is the most commonly used wavelength, and recent studies have shown that light in the second near-infrared region (1000–1700 nm) has deeper tissue penetration and tolerance [91]. The conversion of electromagnetic energy into heat is achieved by photothermal agents (PAT). An excellent PAT is characterized by strong light absorption, strong photothermal conversion ability, stability, good biocompatibility, and the ability to turn off PTT in the non-treatment stage [92]. Near-infrared PAT include polyaniline [93], copper sulfides (CuS) NPs [94], as well as inorganic NPs, such as gold-based NPs [95]. Inorganic NPs show high photothermal conversion efficiency but relatively poor biocompatibility and toxicity [95], while liposome-based material and organic-conjugated polymers exhibit relatively better biocompatibility [96].

As PTT cannot directly kill tumor cells but acts as an adjuvant at about 45 °C, Huang et al. [97] developed a symbiotic mild photothermal-sensitized immunotherapy (SMPAI) and put forward a hypothesis of a synergistic effect by combining mild PTT with immunotherapy. They encapsulated a photothermal and photodynamic therapy agent (IR820) and an anti-PD-L1 antibody into a lipid gel composed of soybean phosphatidylcholine (SPC) and glycerol dioleate (GDO), which undergoes a reversible gel-to-sol transition for the controllable release of aPD-L1 and enhanced infiltration of T cells into tumor under manually controlled NIR irradiation. Overall, mild PTT can activate the systemic immune response, increase the number of TILs, and increase the expression of PD-L1 in tumor cells.

To specifically target CD44, Yasothamani et al. [93] designed a conjugation of hyaluronan (HA)−polyaniline (PANi)−imiquimod (R837), denoted as HA-PANi/R837. The high photothermal conversion efficiency of PANi means that it has a significant thermal ablation effect in solid tumor. HA is a targeting ligand activating CD44 with high biosafety [98], which compensates for the poor targeting and low cellular uptake of PANi. R837 is a toll-like receptor 7 agonist, acting as an immunomodulator. In the TNBC model, HA-PANi/R837 directly killed a part of cancer cells through thermal ablation, induced the production of tumor-associated antigens, and activated immune response. Ultimately, the combination of HA-PANi/R837 and anti-CTLA-4 antibody shows enhanced immunotherapy effect and played a synergistic antitumor effect. For research on PTT, it is clear that it has a certain tumor-killing effect, immunostimulatory effect, and NPs-based PTT can produce NPs-mediated antigen capture [99].

To suppress recurrence and metastasis of TNBC, Cheng et al. [94] designed a biomimetic nanoplatform AM@DLMSN@CuS/R848, based on dendritic large-pore mesoporous silica nanoparticles (DLMSNs) loaded with CuS NPs, immune adjuvant R848 (resiquimod), TNBC cell membrane, and AUNP-12 (a PD-1/PD-L1 peptide inhibitor). AUNP-12 conjugated to the outer TNBC cell membrane by benzoic-imide bond readily released from the nanoparticle in the weakly acidic pH of tumor microenvironment. This nanocarrier exhibits targeted TNBC delivery, high photothermal efficiency of CuS NPs, photothermal-triggered release of R848, and pH-responsive release of AUNP-12, which together lead to strong antitumor efficacy and enhanced therapy against metastatic TNBC.

#### 2.3.2. Immunotherapy Combined with PDT

PDT induces chemical cytotoxic effects to tumor cells on the generation of reactive oxygen species (ROS) [100]. Light, photosensitizer, and molecular oxygen are three indispensable elements for PDT. Light wavelengths in visible (400–700 nm) and near-infrared ranges (700–1350 nm) are commonly used. The photosensitizer absorbs photons and transforms from ground singlet state to excited singlet state, which afterwards generates excited triplet state and relaxation by undergoing intersystem crossing, accompanied by energy emitted as fluorescence, heat, and/or other photophysical energy [92]. The tetrapyrrole structure is extremely common in photosensitizer, and it is related to the photon absorption ability and the ability to convert the relatively low active triplet ground-state molecular oxygen (^3^O_2_) into more active singlet oxygen (^1^O_2_) [101]. Because oxygen content in TME is low (as TME is in a hypoxic state), it is challenging to provide enough molecular oxygen at tumor site for oxygen-dependent PDT.

Combining PDT with immunotherapy could play a synergistic effect. The release of ROS or PDT-induced exposure and/or release of damage-associated molecular patterns (DAMPs) would stimulate the body’s immune system. The increase in immunogenicity induces the maturation of DCs and activation of cytotoxic T lymphocytes to increase the number of TILs [102]. In addition to the immune response caused by PDT itself, the combination of PDT with ICB also plays a synergistic effect in preclinical research. Zhang et al. [87] proved that BMS-202 NPs in combination with Ce6 NPs exhibited a better antitumor and antimetastatic effects. Chung et al. [103] designed smart multifunctional nanoparticle cluster, FM@VP, which combined co-assembly of a nanocomplex formed by a functional polysaccharide fucoidan and a bioreducible polyamidoamine dendrimer, and MnO_2_ NPs encapsulated with a photosensitizer verteporfin. It was able to target P-selectin-overexpressed TNBC. Moreover, due to the high concentration of glutathione, FM@VP clusters would rapidly disintegrate in tumor. The released verteporfin from the clusters enhanced PDT and inhibited yes-associated protein (involved in tumor development), which weakened tumor-mediated immunosuppression. Meanwhile, MnO_2_ NPs could efficiently convert H_2_O_2_ into oxygen in TME, reducing the adverse impression caused by low-oxygen environments [104]. This discovery provided a powerful strategy for synergistic tumor targeting, PDT and immunotherapy.

#### 2.3.3. Immunotherapy Combined with SDT

SDT is a non-invasive therapeutic modality based on ultrasound, which is more acceptable to patients than chemotherapy and radiotherapy. Low-intensity focused ultrasound is commonly used to activate sonosensitizers [105]. Compared with PTT and PDT, the tissue penetration ability of SDT is extremely powerful, thus therapeutic effect for tumors in deep positions is more significant [106,107]. Meanwhile, SDT generates many types of ROS, not only ^1^O_2_, but also hydroxyl (•OH) and superoxide radicals (•O_2_^−^) [108]. Its functions include direct tumor cells killing, and indirect tumor-specific immunity activation through damaging blood vessels or inhibiting the regeneration of tumor tissues, et al. Sonosensitizers, including organic molecules (such as porphyrin derivatives), inorganic nanomaterials (such as TiO2, ZnO) as well as the hybrid ones, have been developed for SDT [109]. To better exert its immunomodulatory effect, the sonosensitizer was delivered through NPs drug-delivery system (DDS) when combined with immune adjuvants in some research.

In TNBC therapy, Chen et al. [110] designed a liposome-encapsulated anganese-protoporphyrin complex (MnP) and modified with folic acid (FA). In the 4T1 model, the multifunctional nanosonosensitizer FA-MnPs exhibited significant effect on both deep-state and superficial tumors. In addition to induce chemical damage to tumor cells, SDT could also exert an effect through immune regulation. For instance, M2 macrophages would develop towards the antitumor M1 phenotype. Similar to PDT, the damage-associated molecular patterns caused by SDT can also stimulate systemic immunity, including the maturation of DCs and the activation of cytotoxic T lymphocytes.

#### 2.3.4. Others

In addition to the direct delivery of immunomodulatory agent to the tumor site through the EPR effect, there are other studies focused on TME targeting. For example, although chemotherapy exert less efficacy to the treatment of TNBC, it also shows immunomodulatory effect on TME. Xu et al. [111] designed a peptide-based, structure-transformable NPs, 2-(Nap)-FFK_Pt-2TPA-ADD_GGGPLGVRG-WKYMVm-mPEG_1000_, for contemporaneous delivery of chemotherapy agent for TNBC, cisplatin and adjudin, as well as WKYMVm—an FPR-1 agonist functions as an immune adjuvant, which synergistically elicit and promote immunogenic cell death for TNBC immunotherapy. In another study, Xu et al. [112] prepared a pueratin nanoemulsion (nanoPue) surface modified with aminoethyl anisamide as targeting ligand to tumor-associated fibroblasts (TAFs). Together with improved solubility and bioavailability, the delivered puerarin significantly deactivate the stromal microenvironment, which synergistically enhances the activity of checkpoint blockade immunotherapy in a TNBC mode when combined with α-PD-L1.

## 3. Conclusions and Perspective

In conclusion, immunotherapy has achieved many promising results in alleviating TNBC. In addition to the classic immune checkpoints PD-1/PD-L1 and CTLA-4, new immune checkpoints are constantly being discovered. At the same time, progress has been made in new treatments, including CAR-T and tumor immunization vaccines. ICIs are commonly used in combination with other therapy, such as chemotherapy, radiotherapy, CAR-T, and tumor vaccine, to obtain synergistic effects for more effective treatment of TNBCs. Moreover, he nano-delivery system integrated with TNBC immunotherapy provides strategy that can achieve precise targeting and reduce off-target effects. Immunotherapy has indeed brought hope to the treatment of TNBC. Although there are problems need to be solved, immunotherapy for TNBC is full of potential.

Finally, we may identify several issues and problems related to the immunotherapy of TNBC. These include: (1) The fundamental understanding of tumor heterogeneity, molecular changes, immunogenomics, and treatment resistance mechanisms in TNBC should be advanced to give better treatments to the patients. (2) The narrow therapeutic window is also a potential problem that needs to be resolved. Although the indication of drugs for monotherapy is clear, it still needs be redefined in combination therapies. Most importantly, because the drug-delivery system of combination therapy is complex, it is more difficult to understand the mechanism. In particular, TME has changed tremendously when it comes to immunotherapy. Most of the research focus on DCs and TILs, but these are definitely not enough. Therefore, immune-regulatory mechanism needs to be further clarified when nanocarrier is involved in immunotherapy. (3) The discovery of new antigens specifically expressed in TNBC cells would facilitate the development of new immunotherapies. (4) For nanocarrier in immunotherapy of TNBC, the safety of nanomaterials is complicated. Particular attention should be paid to inorganic materials with poor biocompatibility and organic nanomaterials with good biocompatibility but strong immunogenicity. (5) Immunoconjugates are effective with minimal toxicity, showing promise for clinical translation. Trodelvy (Sacituzumab govitecan), an antibody-drug conjugate consisting of an antibody Sacituzumab targeting the Trop-2 protein (found in more than 90% of TNBC), and coupled to chemotherapy drugs 7-ethyl-10-hydroxycamptothecin (SN-38, a topoisomerase I inhibitor to interfere with the replication of cancer cells) with a hydrolysable linker, is a first-in-class medicine for advanced TNBC therapy.

## Figures and Tables

**Table 1:** Status of clinical trials with immune checkpoint blockade reagents for the treatment of TNBC [30].

Agent	Target	ClinicalTrials.Gov Identifier	Combinatorial Agent(s)	Phase	Recruitment Status
Atezolizumab	PD-L1	NCT02530489	Nab-paclitaxe	Phase II	Active, not recruiting
Pembrolizumab	PD-1	NCT02622074	Nab-paclitaxel + Doxorubicin + Cyclophosphamide, Nab-paclitaxel + Doxorubicin + Cyclophosphamide + Carboplatin, Doxorubicin + Cyclophosphamide + Carboplatin + Paclitaxel	Phase I	Completed
Pembrolizumab	PD-1	NCT02734290	Paclitaxel, Capecitabine	Phase I Phase II	Active, not recruiting
Pembrolizumab	PD-1	NCT02768701	Cyclophosphamide	Phase II	Active, not recruiting
Pembrolizumab	PD-1	NCT02977468	Intraoperative radiation therapy (IORT)	Phase I	Recruiting
Pembrolizumab	PD-1	NCT02981303	Imprime PGG	Phase II	Completed
Pembrolizumab	PD-1	NCT03012230	Ruxolitinib Phosphate	Phase I	Recruiting
Pembrolizumab	PD-1	NCT03036488	Carboplatin + Paclitaxel + Doxorubicin or Epirubicin + Cyclophosphamide + Granulocyte colony-stimulating factor (G-CSF)	Phase III	Active, not recruiting
Atezolizumab	PD-L1	NCT03125902	Paclitaxel	Phase III	Active, not recruiting
Atezolizumab	PD-L1	NCT03164993	Pegylated liposomal doxorubicin, Cyclophosphamide	Phase II	Recruiting
Durvalumab	PD-L1	NCT03199040	Neoantigen DNA vaccine	Phase I	Active, not recruiting
Atezolizumab	PD-L1	NCT03206203	Carboplatin	Phase II	Active, not recruiting
Atezolizumab	PD-L1	NCT03281954	Paclitaxel + Carboplatin, Doxorubicin + Cyclophosphamide or Epirubicin + Cyclophosphamide	Phase III	Active, not recruiting
Atezolizumab	PD-L1	NCT03371017	Gemcitabine + Capecitabine or Carboplatin	Phase III	Recruiting
Atezolizumab	PD-L1	NCT03424005	Nab-paclitaxel, Nab-paclitaxel + Tocilizumab, Sacituzumab Govitecan, Ipatasertib, Landiratuzumab vedotin (SGN-LIV1A), Selicrelumab + Bevacizumab, Chemo (Gemcitabine + Carboplatin or Eribulin)	Phase I Phase II	Recruiting
Nivolumab	PD-1	NCT03487666	Capecitabine	Phase II	Active, not recruiting
Atezolizumab	PD-L1	NCT03498716	Chemo (Paclitaxel, Dose-dense Doxorubicin or dose-dense Epirubicin), Cyclophosphamide	Phase III	Recruiting
Pembrolizumab	PD-1	NCT03639948	Carboplatin + Docetaxel + Pegfilgrastim	Phase II	Recruiting
Durvalumab	PD-L1	NCT03742102	Paclitaxel, Paclitaxel + Capivasertib, Paclitaxel + Oleclumab, Trastuzumab deruxtecan, Datopotamab deruxtecan	Phase I Phase II	Recruiting
Pembrolizumab	PD-1	NCT03752723	Cyclophosphamide + efineptakin alfa (GX-I7)	Phase I Phase II	Recruiting
Atezolizumab	PD-L1	NCT03756298	Capecitabine	Phase II	Recruiting
Durvalumab	PD-L1	NCT03801369	Olaparib	Phase II	Recruiting
Nivolumab	PD-1	NCT03818685	Ipilimumab	Phase II	Recruiting
Atezolizumab	PD-L1	NCT03853707	Ipatasertib + Carboplatin	Phase I Phase II	Suspended
Pembrolizumab	PD-1	NCT04095689	Docetaxel + Interleukin-12 gene therapy, Docetaxel + NG-monomethyl-L-arginine (L-NMMA)	Phase II	Recruiting
Camrelizumab	PD-1	NCT04129996	Nab-paclitaxel + famitinib	Phase II	Recruiting
Atezolizumab	PD-L1	NCT04148911	Nab-paclitaxel	Phase III	Recruiting
Atezolizumab	PD-L1	NCT04177108	Ipatasertib	Phase III	Active, not recruiting
Pembrolizumab	PD-1	NCT04191135	Carboplatin + Gemcitabine, Carboplatin + Gemcitabine + Olaparib	Phase II Phase III	Active, not recruiting
Camrelizumab	PD-1	NCT04331067	Nivolumab + Paclitaxel + Carboplatin	Phase I Phase II	Recruiting
Camrelizumab	PD-1	NCT04335006	Nab-paclitaxel + Apatinib, Nab-paclitaxel	Phase III	Recruiting
Camrelizumab	PD-1	NCT04481763	Radiotherapy	Phase I Phase II	Recruiting
Tiragolumab and Atezolizumab	PD-L1	NCT04584112	Nab-paclitaxel, Nab-paclitaxel + Carboplatin + Doxorubicin + Cyclophosphamide + G-CSF or Granulocyte-macrophage colony-stimulating factor (GM-CSF), Nab-paclitaxel + Doxorubicin + Cyclophosphamide + G-CSF + GM-CSF	Phase I	Recruiting
Camrelizumab	PD-1	NCT04613674	Chemotherapy	Phase III	Recruiting
Camrelizumab	PD-1	NCT04676997	Nab-paclitaxel + Epirubicin + Cyclophosphamide	Phase II	Recruiting
Pembrolizumab	PD-1	NCT04683679	Olaparib + Radiation, Radiation	Phase II	Recruiting

## Data Availability

Not applicable.
